# Association of tinnitus and self-reported systemic arterial hypertension: a retrospective study

**DOI:** 10.1590/2317-1782/20212021236en

**Published:** 2022-11-04

**Authors:** Camila Soares Carneiro, Raquel Elpidio Pinheiro da Silva, Jerusa Roberta Massola de Oliveira, Maria Fernanda Capoani Garcia Mondelli

**Affiliations:** 1 Hospital de Reabilitação de Anomalias Craniofaciais - HRAC, Universidade de São Paulo - USP - Bauru (SP), Brasil.; 2 Programa de Pós-graduação, Universidade de São Paulo - USP - Bauru (SP), Brasil.

**Keywords:** Tinnitus, Hypertension, Hearing Loss, Hearing, Comorbidity

## Abstract

**Purpose:**

To describe data on hearing loss, systemic arterial hypertension and tinnitus of individuals, and to verify the association between self-reported systemic arterial hypertension and tinnitus, as well as to correlate other variables present in the sample: hearing loss and tinnitus, age and tinnitus and age and systemic arterial hypertension.

**Methods:**

Quantitative, descriptive and inferential, retrospective research with data collection from 473 medical records of adults and elderly people treated between 2008 and 2018. Selected were information on age, gender, result of pure tone audiometry, tinnitus, tinnitus type and frequency, presence of SAH and use of medication to control the disease.

**Results:**

No association was found between systemic arterial hypertension and tinnitus or between hearing loss and tinnitus and between age and tinnitus, however, an association was observed between age and systemic arterial hypertension using the Chi - Square test. The most common type of tinnitus was wheezing and most individuals who reported feeling more than one type of tinnitus were hypertensive.

**Conclusion:**

The results found and the literature suggest that systemic arterial hypertension may be an additional factor or an aggravating factor of preexisting factors in the generation of tinnitus, but not the primary cause.

## INTRODUCTION

Tinnitus is defined as an auditory sensation without an external sound stimulus or meaning, which can be experienced as an unpleasant experience, and can impact quality of life^(1)^. It can be continuous, intermittent or sporadic and can be felt in one of the ears, in both ears, or “in the head”^(2)^. Tinnitus is classified as objective when the cause is mechanical in an area attached to the ear or subjective when there are multiple causes or there is no exact cause that triggers it^(3)^.

There are several risk factors for tinnitus^(3)^. In addition, hearing impairment may be associated with chronic non-communicable diseases such as Systemic Arterial Hypertension (SAH)^(4)^, which is characterized by elevation and maintenance of blood pressure (BP), with systolic pressure equal to or greater than 140 mmHg and diastolic blood pressure equal to or greater than 90 mmHg^(5)^. It can also be classified as stage 1 with systolic blood pressure of 130 to 139 mm Hg or diastolic BP of 80 to 89 mm Hg and stage 2 with systolic blood pressure of 140 mm Hg or more and diastolic BP of 90 mm Hg or even more^(4)^. 

It is a multifactorial clinical condition that affects organs such as the heart, kidneys, blood vessels and the brain^(5)^, and the increase in the prevalence of SAH may be associated with aging, which may be the main determining factor of this increase since many of these factors are modifiable factors such as smoking, alcoholism and physical inactivity^(6)^.

Furthermore, the increase in BP can cause hemorrhage in the inner ear, which, like other parts of the body^(5)^, receives blood supply from the anterior inferior cerebellar artery which divides and supports other branches of the ear and may culminate in sudden or progressive hearing loss^(7)^. In addition, the literature points out that SAH can be one of the causes or aggravating causes of tinnitus, and the alteration of the blood microcirculation of the inner ear is an aggravating factor, as well as the ototoxicity caused by antihypertensive drugs and the perception of noise generated by blood flow due to bony dehiscence of the carotid artery canal^(7,8)^.

The influence of SAH on the onset or worsening of tinnitus is still unclear^(7,8)^.A systematic review that also performed a meta-analysis of the research found on the onset of tinnitus associated with SAH revealed an uncertain relationship. Of the 19 studies found, only eight showed a statistically significant association between tinnitus and SAH^(9)^.

Therefore, the present research started from the hypothesis that SAH can act as a primary or an aggravating factor, together with other organic or behavioral factors in the generation of tinnitus. Therefore, the objectives of the present study were to describe data on hearing loss, systemic arterial hypertension and tinnitus of individuals, and to verify the association between SAH and tinnitus, as well as to correlate other variables present in the sample: hearing loss and tinnitus, age and tinnitus and between age and SAH.

## METHODS

The present research was approved by the Research Ethics Committee of the institution under CAAE 85057518.7.0000.5441, in accordance with the ethical principles of Resolution No. 466/12. Quantitative, descriptive and retrospective inferential with data collection from 473 medical records of adults and elderly people attended between 2008 and 2018, considering the following inclusion criteria: adults aged between 18 and 59 years and elderly people from 60 years of age ^(10)^, hearing thresholds within the normal range or bilateral sensorineural hearing loss^(11)^, bilateral “A” type tympanometric curves^(12)^, not presenting a history of middle ear alteration, which was verified in the otorhinolaryngologist's progress sheet, not being User of an Individual Sound Amplification Device (HA) at the time of anamnesis or initial evaluation for individuals with hearing loss and the presence of SAH that was self-reported by the individuals so that the blood pressure measurement was not verified.

Since the present is a retrospective study, it was requested not to use the informed consent because most of the information collected was from old medical records, resulting in the impossibility of contacting and inviting patients to sign the term due to various reasons, such as changing contact data not informed, discharge and death.

The collection included sociodemographic and audiological data, including tinnitus data such as type and frequency, presence of self-reported SAH and use of medication to control the disease.

Data analysis was performed using the Excel program. The treatment of variables was carried out through descriptive statistics using the relative frequency, and was presented through tables and graphs. The associations between the variables collected were analyzed using the chi - square test.

## RESULTS

A total of 473 medical records were collected from patients who met the inclusion criteria. Of these, 283 (59.8%) were female and 190 (40.2%) were male. The mean age of the individuals was 62.3 ± 17.1 years.

Of the total of 473 individuals, 346 (73.2%) had hearing loss. The tinnitus complaint was reported by 255 (53.9%) subjects and the presence of SAH was reported by 208 (44.0%) subjects (Table 1).

**Table 1:** Prevalence of hearing loss, tinnitus and systemic arterial hypertension of the research subjects

Individuals (n=473)	no	%
Norm -listener	127	26.8
Hearing Loss:		
Light	118	34.1
Moderate	201	58.1
Severe	26	7.5
Deep	1	0.3
Buzz:		
Gift	255	53.9
Absent	218	46.1
HAS:		
Gift	208	44.0
Absent	265	56.0

Subtitle Chi- square Test

**Caption:** SAH = Systemic arterial hypertension; n = number of subjects; % = relative frequency.

Of the individuals who claimed to have SAH, only one (0.5%) reported that they were not using medication to control the disease.

Twenty-four (9.4%) subjects reported feeling more than one type of tinnitus, however, the feeling of a single tinnitus prevailed in 231 (90.6%) individuals. Of the total number of subjects who reported more than one type of tinnitus, 16 (66.7%) were hypertensive.

Regarding location, 166 (65.1%) reported bilateral tinnitus, 77 (30.2%) reported unilateral tinnitus and 14 (5.5%) reported tinnitus felt “in the head.”

Regarding the frequency, the continuous sensation of tinnitus was reported by 126 (49.4%) subjects, frequent by 57 (22.3%) and sporadic by 72 (28.2%) subjects.

**Figure 1:**
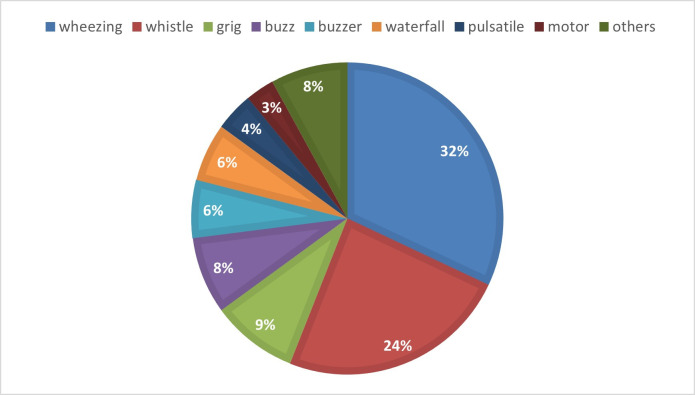
Distribution of types of tinnitus reported by research subjects

The type of tinnitus most frequently reported was hissing (32.5%), on the other hand, 21 (7.7%) subjects reported feeling less common types, such as turbine sound, butterfly, rain, crackling, among others, be it this sensation alone or accompanied by other types. It is important to note that, due to some individuals reporting more than one type of tinnitus, the total number of tinnitus types was 274 (100%) (Figure 1).

When verifying the association between SAH and tinnitus, no statistically significant difference was found in the relationship between the variables in the population of the present study (Table 2).

**Table 2:** Analysis of the variables systemic arterial hypertension and tinnitus

Variable	no buzz		with buzz		p value
	no	%	no	%	
Norm -listener	60	47.2	67	52.8	0.840
Hearing Loss	158	45.7	188	54.3	

Subtitle Chi- square Test

When analyzing the relationship between hearing loss and tinnitus, no difference was found for these variables (p>0.05), however, it was observed that of the 187 (100%) individuals diagnosed with hearing loss who reported feeling tinnitus, 103 (54.8%) were classified as having moderate hearing loss, followed by 74 (39.4%) as mild, 11 (5.9%) as severe and none as profound (Table 3).

**Table 3:** Analysis of the variables hearing loss and tinnitus

Variable	Category	no buzz		with buzz		p value
		no	%	No	%	
Hypertension	No	127	47.9	138	52.1	0.417
	Yes	91	43.8	117	56.3	

Subtitle Chi- square Test

By dividing the individuals into two groups according to age, it was possible to verify the relationship between age and the occurrence of SAH and tinnitus in the Adult Group up to 59 years (Table 4), with a total of 171 (36.1%) subjects, and in the Elderly Group from 60 years old (Table 5), with a total of 302 (63.8%) subjects.

**Table 4:** Analysis of age and zombie variables

Variable	no buzz		with buzz		p value
	no	%	no	%	
Elderly	138	45.7	164	54.3	0.895
Adults	80	46.8	91	53.2	

Subtitle Chi- square Test

**Table 5:** Analysis of age and systemic arterial hypertension variables

Variable	Hypertension				p value
	Yes		No		
	no	%	no	%	
Elderly	172	57.0	130	43.0	<0.001*
Adults	36	21.1	135	78.9	

Subtitle: Chi- square Test

**Caption:** * = Statistically significant value.

There was no relationship between age and the occurrence of tinnitus (p>0.05), however, an association was observed between the variables SAH and age of the individuals (p<0.05).

## DISCUSSION

In the present study, no association was observed between tinnitus and SAH, and this result corroborates another study^(13)^ in which one of its objectives was to analyze the prevalence of tinnitus complaints and the possible associations with hearing loss, diabetes mellitus and arterial hypertension in elderly people, and did not find an association between tinnitus and some cardiovascular risk factors, including SAH. This finding may have been influenced by the fact that most individuals in the study used medication to control SAH.

However, the result found in the present research is in agreement with another study that aimed to determine the prevalence of tinnitus in hypertensive patients undergoing treatment with antihypertensive drugs whose results were a high prevalence of tinnitus in individuals who had staged hypertension stage 1 or 2^(14)^. This difference in the findings can be explained by the fact that in the present study, we did not have information regarding the stage of SAH since the presence or absence of hypertension was self-reported, and the SAH measurement was not actually performed. It is therefore suggested that further research be carried out to correlate the type of stage of SAH present in hypertensive patients by verifying the measurement of blood pressure and its influence on the appearance of the tinnitus symptom.

Furthermore, in a systematic review^(15)^, it was found that tinnitus worsens when there is an abrupt increase or decrease in BP, and the control and restoration of BP lead to an improvement in this symptom. Therefore, it can be inferred that SAH may be a cofactor for the generation of tinnitus or an aggravating factor of preexisting alterations, but not its primary cause. ^(7-15-9)^

The type of tinnitus reported by the individuals in the present research were the most diverse., Most frequently referred to wheezing, followed by the whistle, and these findings corroborate the study^(7)^ which also found that the complaint of feeling of more than one type of tinnitus is more common in hypertensive individuals. This result was also observed in the present research, because of the total of 24 (100%), the prevalence was 16 (66.7%) hypertensive individuals who reported feeling more than one type of tinnitus.

Most of the records collected were from female patients. The prevalence of this gender was also observed in a Brazilian study^(7)^, in two Korean studies^(16,17)^, and in a study carried out in Pakistan^(14)^, while in an Italian study^(18)^ the male gender predominated. In another study, no difference was observed between genders^(13)^. The divergence in the predominance of gender in the various studies may be influenced by the different distribution of the prevalence of organic and behavioral risk factors which occur due to cultural and lifestyle differences in the population of each country. For the present research, the predominance of the female gender may be related to the fact that this gender is one of the risk factors for both SAH and tinnitus^(5-19)^.

A higher prevalence of SAH among elderly patients was also observed in the present study, a result that corroborates other studies^(7-20)^. This finding presents a reflection for future research regarding the relationship between the variables age and presence of SAH associated with tinnitus. It is also suggested that further research be carried out to analyze the association of two or more risk factors in the generation of tinnitus.

## CONCLUSION

There was no association between tinnitus and self-reported SAH, a result that may have been influenced by the use of antihypertensive drugs, as well as the type of stage of SAH. The same occurred in the relationship between hearing loss and tinnitus and the relationship between age and tinnitus.

The results found and the analysis of the literature suggest that SAH may be a cofactor or an aggravating factor of preexisting factors in the generation of tinnitus, but not the primary cause.
