# Supplementary material for: Association of tinnitus and self-reported systemic arterial hypertension: a retrospective study
Source: Codas. 2022 Nov 4;34(6):e20210236. doi: 10.1590/2317-1782/20212021236en (PMC9886292; doi:10.1590/2317-1782/20212021236en)
Supplement: Translation

# Translation: Associação do zumbido e a hipertensão arterial sistêmica autorreferida: estudo retrospectivo

**DOI:** 10.1590/2317-1782/20212021236pt

**Authors:** Camila Soares Carneiro, Raquel Elpidio Pinheiro da Silva, Jerusa Roberta Massola de Oliveira, Maria Fernanda Capoani Garcia Mondelli

**Affiliations:** 1 Hospital de Reabilitação de Anomalias Craniofaciais - HRAC, Universidade de São Paulo - USP - Bauru (SP), Brasil.; 2 Programa de Pós-graduação, Universidade de São Paulo - USP - Bauru (SP), Brasil.

**Keywords:** Zumbido, Hipertensão, Perda Auditiva, Audição, Comorbidade

## Abstract

**Objetivo:**

Descrever dados da perda auditiva, da hipertensão arterial sistêmica e do zumbido dos indivíduos, e verificar a associação entre hipertensão arterial sistêmica autorreferida e zumbido, bem como correlacionar outras variáveis presentes na amostra: perda auditiva e zumbido, idade e zumbido e idade e hipertensão arterial sistêmica.

**Método:**

Pesquisa quantitativa, descritiva e inferencial, retrospectiva com coleta de dados de 473 prontuários de adultos e idosos atendidos entre os anos 2008 e 2018. Selecionadas informações sobre idade, gênero, resultado da audiometria tonal liminar, zumbido, tipo e frequência do zumbido, presença de HAS e uso de medicamento para controle da doença.

**Resultados:**

não foi encontrada associação entre hipertensão arterial sistêmica e zumbido ou entre perda auditiva e zumbido e entre idade e zumbido, todavia foi observada associação entre idade e hipertensão arterial sistêmica, por meio do teste Qui Quadrado. O tipo de zumbido mais comum foi o chiado e a maioria dos indivíduos que referiram sentir mais de um tipo de zumbido eram hipertensos.

**Conclusão:**

os resultados encontrados e a literatura sugerem que a hipertensão arterial sistêmica pode ser um fator adicional ou um agravante de fatores preexistentes na geração do zumbido, porém não a causa primária.

## INTRODUÇÃO

O zumbido é definido como uma sensação auditiva sem um estímulo ou significado sonoro externo, que pode ser vivido como uma experiência desagradável, podendo impactar na qualidade de vida^(1)^. Pode ser contínuo, intermitente ou esporádico e ser sentido em uma das orelhas, em ambas, ou “na cabeça”^(2)^. Classifica-se o zumbido como objetivo, quando a causa é mecânica em uma área anexa ao ouvido, ou subjetivo, quando há múltiplas ou não há uma causa exata que o desencadeie^(3)^. 

Existem diversos fatores de risco para o aparecimento do zumbido^(3)^, além disso, o comprometimento auditivo pode estar associado a doenças crônicas não transmissíveis, à exemplo da Hipertensão Arterial Sistêmica (HAS)^(4)^, que é caracterizada pela elevação e sustentação da pressão arterial (PA), com pressão sistólica igual ou maior a 140 mmHg e diastólica igual ou maior a 90 mmHg^(5)^. A qual também podemos classificá-la em estágio 1, com pressão arterial sistólica de 130 a 139 mm Hg ou PA diastólica de 80 a 89 mm Hg e estágio 2 com pressão sistólica de 140 mm Hg ou mais e PA diastólica de 90 mm Hg ou até mais^(4)^.

É uma condição clínica multifatorial que afeta órgãos como coração, rins, vasos sanguíneos e encéfalo^(5)^, e o aumento da prevalência da HAS pode estar associado ao envelhecimento, podendo este ser o principal fator determinante desse acréscimo uma vez que muitos desses fatores são modificáveis como o tabagismo, alcoolismo e sedentarismo^(6)^.

Além disso, o aumento da PA pode causar hemorragia a nível de orelha interna que assim como outras partes do corpo^(5)^, recebe suprimento de sangue provido da artéria cerebelar inferior anterior, que se divide e dá suporte a outros ramos da orelha, podendo culminar em perdas auditivas súbitas ou progressivas^(7)^. Somado a isso, a literatura aponta que a HAS pode ser uma das causas geradoras ou agravantes do zumbido, sendo a alteração da microcirculação sanguínea da orelha interna um fator agravante, como também, a ototoxicidade causada pelos medicamentos anti-hipertensivos e a percepção de ruído gerado pelo fluxo sanguíneo devido a deiscência óssea do canal da artéria carótida^(7,8)^.

A influência da HAS no surgimento ou agravamento do zumbido ainda não está clara^(7,8)^. Uma revisão sistemática que também realizou meta-análise das pesquisas encontradas sobre o aparecimento do zumbido associado à HAS revelou uma relação incerta. Das 19 pesquisas encontradas, somente oito apresentaram associação estatisticamente significante entre zumbido e HAS^(9)^.

Sendo assim, a presente pesquisa partiu da hipótese de que a HAS pode agir como fator primário ou um agravante, junto a outros fatores orgânicos ou comportamentais, na geração do zumbido. Portanto, os objetivos desse estudo, foram descrever dados da perda auditiva, da hipertensão arterial sistêmica e do zumbido dos indivíduos, e verificar a associação entre HAS e zumbido, bem como correlacionar outras variáveis presentes na amostra: perda auditiva e zumbido, idade e zumbido e entre idade e HAS.

## MÉTODO

Trata-se de pesquisa aprovada pelo Comitê de Ética em Pesquisa da instituição, sob o CAAE 85057518.7.0000.5441, de acordo com os princípios éticos da Resolução n° 466/12. Quantitativa, descritiva e inferencial retrospectiva com coleta de dados de 473 prontuários de adultos e idosos atendidos entre os anos 2008 e 2018, contemplando os seguintes critérios de inclusão: adultos na faixa etária de 18 a 59 anos e idosos a partir de 60 anos^(10)^_,_ limiares auditivos dentro dos padrões de normalidade ou perda auditiva sensorioneural bilateral^(11)^_,_ curvas timpanométricas do tipo ”A” bilateral^(12)^, não apresentar histórico alteração de orelha média, que foi verificado na ficha de evolução do otorrinolaringologista, não ser usuário de Aparelho de Amplificação Sonora Individual (AASI) no momento da anamnese ou avaliação inicial para os indivíduos com perda auditiva e a presença da HAS que foi autorreferida pelos indivíduos, de modo que não foi verificado a medida de pressão arterial.

Por se tratar de um estudo retrospectivo, foi solicitado a não utilização do TCLE devido grande parte das informações coletadas serem de prontuários antigos, acarretando na impossibilidade de contatar e convidar os pacientes para assinatura do termo devido diversos motivos, como alteração de dados de contato não informada, recebimento de alta e óbito.

A coleta contemplou dados sociodemográficos, audiológicos, incluindo dados do zumbido como tipo e frequência, presença de HAS autorreferida e uso de medicamento para controle da doença.

A análise dos dados foi realizada com o auxílio do programa Excel. O tratamento das variáveis foi realizado através de estatística descritiva, com uso da frequência relativa, e sua apresentação por meio de tabelas e gráficos. As associações entre as variáveis coletadas foram analisadas através do teste Qui-quadrado.

## RESULTADOS

Foram coletados 473 prontuários de pacientes que atenderam aos critérios de inclusão. Destes, 283 (59,8%) do gênero feminino e 190 (40,2%) do gênero masculino. A média de idade dos indivíduos foi de 62,3 ± 17,1 anos.

Do total de 473 indivíduos, 346 (73,2%) apresentaram perda auditiva. A queixa de zumbido foi referida por 255 (53,9%) sujeitos e a presença de HAS foi referida por 208 (44,0%) indivíduos (Tabela 1).

**Tabela 1:** Prevalência da perda auditiva, zumbido e hipertensão arterial sistêmica dos sujeitos da pesquisa

Indivíduos (n=473)	n	%
Normo-ouvinte	127	26,8
Perda auditiva:		
Leve	118	34,1
Moderada	201	58,1
Severa	26	7,5
Profunda	1	0,3
Zumbido:		
Presente	255	53,9
Ausente	218	46,1
HAS:		
Presente	208	44,0
Ausente	265	56,0

Teste estatístico Qui- Quadrado

**Legenda:** HAS = Hipertensão arterial sistêmica; n = número de sujeitos; % = frequência relativa.

Dos indivíduos que afirmaram ter HAS, somente um (0,5%) referiu que não estava utilizando medicamento para controlar a doença.

Vinte e quatro (9,4%) sujeitos referiram sensação de mais de um tipo de zumbido, no entanto, prevaleceu a sensação de um único zumbido em 231 (90,6%) indivíduos. Do total de sujeitos que referiram mais de um tipo de zumbido, 16 (66,7%) eram hipertensos.

Quanto à localização, foram observados 166 (65,1%) relatos de zumbido bilateral, 77 (30,2%) de zumbido unilateral e 14 (5,5%) relatos de zumbido sentido “na cabeça”.

Quanto à frequência, a sensação contínua do zumbido foi referida por 126 (49,4%) sujeitos, frequente por 57 (22,3%) e esporádico por 72 (28,2%) indivíduos.

O tipo de zumbido mais frequentemente relatado foi o chiado (32,5%), por outro lado, 21 (7,7%) sujeitos referiram sentir tipos menos comuns, como som de turbina, borboleta, chuva, estalo, entre outros, seja esta sensação a única ou acompanhada de outros tipos. É importante ressaltar que, devido a alguns indivíduos terem relatado mais de um tipo de zumbido, o n total do tipo de zumbido foi de 274 (100%) (Figura 1).

**Figura 1:** Distribuição dos tipos de zumbido relatados pelos sujeitos da pesquisa

Ao verificar a associação entre HAS e zumbido, não foi encontrada diferença estatisticamente significativa da relação entre as variáveis na população da presente pesquisa (Tabela 2).

**Tabela 2:** Análise das variáveis hipertensão arterial sistêmica e zumbido

Variável	Sem zumbido		Com zumbido		Valor de p
	n	%	n	%	
Normo-ouvinte	60	47,2	67	52,8	0,840
Perda auditiva	158	45,7	188	54,3	

Teste estatístico Qui- Quadrado

Ao realizar a análise da relação entre perda auditiva e zumbido, também não foi encontrada diferença para estas variáveis (p>0,05), no entanto, foi observado que dos 187 (100%) indivíduos diagnosticados com perda auditiva que referiram sentir zumbido, 103 (54,8%) foram classificados com perda auditiva de grau moderado, seguido de 74 (39,4%) com grau leve, 11 (5,9%) com grau severo e nenhum com grau profundo (Tabela 3).

**Tabela 3:** Análise das variáveis perda auditiva e zumbido

Variável	Categoria	Sem zumbido		Com zumbido		Valor de p
		n	%	N	%	
Hipertensão	Não	127	47,9	138	52,1	0,417
	Sim	91	43,8	117	56,3	

Teste estatístico Qui- Quadrado

Ao dividir os indivíduos em dois grupos de acordo com a faixa etária, foi possível verificar a relação entre a idade e a ocorrência da HAS e do zumbido no Grupo Adulto até 59 anos (Tabela 4), com o total de 171 (36,1%) sujeitos, e no Grupo Idoso a partir de 60 anos (Tabela 5), com o total de 302 (63,8%) sujeitos.

**Tabela 4:** Análise das variáveis idade e zumbido

Variável	Sem zumbido		Com zumbido		Valor de p
	n	%	n	%	
Idosos	138	45,7	164	54,3	0,895
Adultos	80	46,8	91	53,2	

Teste estatístico Qui- Quadrado

**Tabela 5:** Análise das variáveis idade e hipertensão arterial sistêmica

Variável	Hipertensão				Valor de p
	Sim		Não		
	n	%	n	%	
Idosos	172	57,0	130	43,0	<0,001 *
Adultos	36	21,1	135	78,9	

Teste estatístico Qui- Quadrado

**Legenda:** * = Valor estatisticamente significativo.

Não foi observada relação entre a idade e a ocorrência de zumbido (p>0,05), no entanto, foi observada associação entre as variáveis HAS e idade dos indivíduos (p<0,05).

## DISCUSSÃO

No presente estudo, não foi observada associação entre zumbido e HAS, e esse resultado corrobora com um estudo^(13)^ em que um dos seus objetivos foi analisar a prevalência da queixa de zumbido e as possíveis associações com perda auditiva, diabetes mellitus e hipertensão arterial em pessoas idosas, que não verificou associação entre zumbido e alguns fatores de risco cardiovasculares, inclusive a HAS. Esse achado, pode ter sido influenciado pelo fato da maioria dos indivíduos desse estudo, utilizarem medicamento para controle da HAS.

Entretanto, o resultado encontrando na presente pesquisa, vai de encontro a outro estudo que teve como objetivo determinar a prevalência do zumbido em pacientes hipertensos em tratamento com anti-hipertensivos, cujo os resultados foram de alta prevalência de zumbido nos indivíduos que tinham hipertensão em estágio 1 ou estágio 2^(14)^. Essa diferença dos achados, pode ser explicada diante do fato de na presente pesquisa, não termos a informação em relação ao estágio da HAS, visto que a presença ou não da hipertensão foi autorreferida, não sendo realizado a medida da HAS de fato. É sugestível então, a realização de mais pesquisas para correlacionar o tipo de estágio da HAS presente nos pacientes hipertensos, através da verificação da medida da pressão arterial e a sua influência no aparecimento do sintoma zumbido.

Além disso, em uma revisão sistemática^(15)^, foi verificado que o zumbido se agrava quando ocorre o aumento ou diminuição abrupta da PA, bem como o controle e restabelecimento da PA, levam a uma melhora desse sintoma. Sendo assim, pode-se inferir que a HAS pode ser um cofator para a geração do zumbido, ou um agravante de alterações preexistentes, mas não sua causa primária ^(7-15- 9)^.

O tipo de zumbido referido pelos indivíduos nessa pesquisa, foram os mais diversos, sendo o mais frequentemente referido o chiado, seguido do apito, e esses achados corroboram com o estudo^(7)^ que também verificou que a queixa de sensação de mais de um tipo de zumbido é mais comum em indivíduos hipertensos. Este resultado também foi observado nesta pesquisa, pois do total de 24 (100%), a prevalência foi de 16 (66,7%) indivíduos hipertensos que referiram sentir mais de um tipo de zumbido.

A maioria dos prontuários coletados foram de pacientes do gênero feminino. A prevalência deste gênero também foi observada em uma pesquisa brasileira^(7)^, em dois estudos coreanos^(16,17)^, e em um estudo realizado no Paquistão^(14)^_,_enquanto que em uma pesquisa italiana^(18)^ apresentou predominância do gênero masculino em sua população. Em outro estudo, não foi observado diferença entre os gêneros^(13)^. A divergência do predomínio de gênero nas diversas pesquisas pode ser influenciada pela diferente distribuição da prevalência dos fatores de risco orgânicos e comportamentais, que ocorrem devido às diferenças culturais e de estilo de vida da população de cada país. Para a presente pesquisa, a predominância do gênero feminino pode estar relacionada ao fato de este gênero ser um dos fatores de risco tanto para a HAS quanto para o zumbido^(5-19)^.

Também foi observado na presente pesquisa, uma maior prevalência da HAS entre os pacientes idosos, resultado esse que corrobora com outros estudos^(7-20)^. E esse achado traz uma reflexão para pesquisas futuras no tocante a relação entre as variáveis idade e presença de HAS associado ao zumbido. Sugere-se também, a realização de novas pesquisas que analisem a associação de dois ou mais fatores de risco na geração do zumbido.

## CONCLUSÃO

Não foi observada associação entre zumbido e HAS autorreferida, resultado esse que pode ter sido influenciado pelo uso de medicamentos anti-hipertensivos, como também, o tipo de estágio da HAS. O mesmo ocorreu na relação entre perda auditiva e zumbido e a relação entre a idade e o zumbido.

Os resultados encontrados e a análise da literatura sugerem que a HAS pode ser um cofator ou um agravante de fatores preexistentes na geração do zumbido, porém não a causa primária.
